# A swift, easy and cheap protocol to evaluate the tooth bleaching *in vitro*

**DOI:** 10.4317/jced.54828

**Published:** 2018-06-01

**Authors:** Klayann-Varejão-de Freitas Penha, Anne-Carolyne-Santos Sousa, Camila-Araújo Oliveira, Raissa-Silva-Bacelar de Andrade, Daniel-Fernando-Pereira Vasconcelos

**Affiliations:** 1 Laboratory of Histological Analysis and Preparation (LAPHIS), Federal University of Piaui, Parnaiba, PI, Brazil

## Abstract

**Background:**

This study aims to develop a protocol that optimizes *in vitro* dental bleaching procedures in a cheap, fast and accessible manner.

**Material and Methods:**

18 bovine incisors were cut and polished in enamel/dentin and submitted to staining in coffee solution during 72 hours. After the standardization and staining of the enamel surfaces, three groups (n = 6) were formed to receive three different gels, WHITENESS (commercial gel); H2O2 - 5% (manipulated gel); CARBOPOL GEL (manipulated gel and without H2O2). The color of the enamel surfaces was evaluated using digital images, obtained with digital camera and controlled luminosity, before and after staining, as well as after each bleaching step.

**Results:**

The average bleaching after 48 hours of procedure was: WHITENESS with 13.6 (± 1.2); H2O2 (5%) with 9.8 (± 1.4) and CARBOPOL GEL with 2.9 (± 0.6). After 72 hours, WHITENESS presented a mean of 15.8 (± 0.7), the H2O2 group (5%) 14.4 (± 1.5) and CARBOPOL GEL 4.6 (± 1.0). After completing 96 hours of whitening, WHITENESS presented an average of 18.3 (± 0.8), H2O2 (5%) 16.7 (± 1.4) and CARBOPOL GEL 7.3 (± 0.8). Our data demonstrated that the protocol development for us can be used to evaluate dental bleaching in a short time, with 96 hours already was possible to detect significant differences, when compared with the longer times of experimental dental bleaching.

**Conclusions:**

The proposed protocol guarantees statistically significant results in 96 hours, confirming the efficacy, cheapness, viability and practicality of the protocol developed in this study.

** Key words:**Enamel, color, aesthetics.

## Introduction

The search for the improvement of dental aesthetics is increasingly present in modern society (1). Satisfaction with dental appearance is strongly influenced by tooth color (2-5). With the increase in aesthetic value and the search for whitening procedures, the research was conducted by techniques and procedures that guarantee results without compromising the health of dental tissues (2), which could affect the subject’s quality of life (6). Increasingly young people and children are worried and dissatisfied with the aesthetic dentistry. Aesthetic problems in childhood and adolescence can significantly influence psychosocial development and interactions with colleagues. In a study in the United States with 2,495 children, 31.6% were dissatisfied with tooth color and 19% of parents shared this dissatisfaction (7). A study carried out in Malaysia showed that 56.2% of 235 patients were not satisfied with the color of the teeth, and 48.1% wanted to perform dental whitening (8).

Tooth whitening is a process that seeks to make the tooth as clear as possible. The color of the teeth is determined by the combined effects of the intrinsic and extrinsic dyes, intrinsic color and especially those associated with the property of dispersion and adsorption of enamel and dentin light and extrinsic colors that tend to form in areas of the teeth which are less accessible for tooth brushing and are often promoted by smoking, dietary intake, the use of certain agents such as chlorhexidine or metal salts (9,10).

Bleaching is performed according to the active agent, especially hydrogen peroxide (H2O2), or one of its precursors, such as carbamide peroxide (11-13). H2O2 acts as an important agent in tooth whitening, releasing free radicals and oxidative chromophore molecules through redox processes. The penetration of oxidative agents into dental structures breaks these chromophore molecules into less complex molecules, generating a clearer dental appearance (14). The chemical reaction of the reagent in contact with an organic matter, including pigments and chromophores, is the chemical basis for tooth whitening (15).

Many are the protocols available for teeth whitening. However, in most of them, the high cost of the equipment, the complexity of the process and the long period to achieve desirable results are disadvantageous factors, requiring alternatives that obtain similar, more practical and cheaper results (16).

Most of the available *in vitro* dental whitening protocols perform the dental whitening procedure at times greater than 96 hours, making them more time consuming (17,18). This study aims to develop a protocol that performs dental bleaching procedures *in vitro* in a cheap, fast and accessible manner.

## Material and Methods

-Specimen preparation

Eighteen bovine incisor teeth that were extracted and randomly selected after being collected and disinfected with chlorhexidine 2%. The teeth were obtained from a local slaughterhouse. Therefore, the approval of the Ethics Committee was not necessary for the present article, since the teeth were destined to the discard.

**Figure 1:**
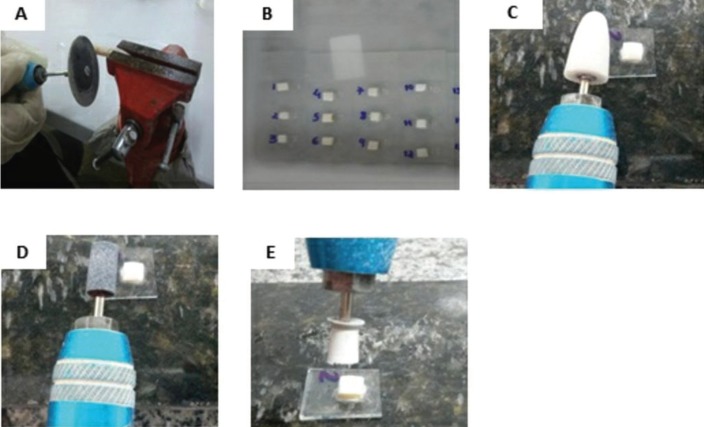
(A) The clamp (Professional Line Number Lathe 3 / METALSUL - TBP05), the teeth were established by the root area, for better manipulation and safety in the accomplishment of dental crown cuts, the crowns were cut in enamel/ dentin measurements of 5 mm of width by 10 mm of length with a cutting disc (22mm x 0.6mm / 223 - Detoriom). (B) The enamel/ dentin specimens were randomly attached with glue (Super Bonder®, Itapevi, SP, Brazil) on glass slides through the dentin, exposing the dental enamel, with 3 specimens on each slide. (C) the surfaces of the enamel were polished using a micro electric motor (LB 100 Beltec) with a thick granular abrasion stone (DhPro). (D) followed by fine granular abrasion stone (DhPro). (E) removing 200μm from the enamel surface to obtain smooth surfaces, completing the polishing with a rubber cup.

With the help of a clamp (Professional Line Number Lathe 3 / METALSUL - TBP05), the teeth were established by the root area, for better manipulation and safety in the accomplishment of dental crown cuts, the crowns were cut in enamel/ dentin measurements of 5 mm of width by 10 mm of length with a cutting disc (22mm x 0.6mm / 223 - Detoriom) as shows in Fig. 1 A,B.

The enamel/ dentin specimens were randomly attached with glue (Super Bonder®, Itapevi, SP, Brazil) on glass slides through the dentin, exposing the dental enamel, with 3 specimens on each slide (Fig. 1B). Then, the surfaces of the enamel were polished using a micro electric motor (LB 100 Beltec) with a thick granular abrasion stone (DhPro) (Fig. 1C), followed by fine granular abrasion stone (DhPro) (Fig. 1D), removing 200μm from the enamel surface to obtain smooth surfaces, completing the polishing with a rubber cup (Fig. 1E).

-Staining of specimen

**Figure 2:**
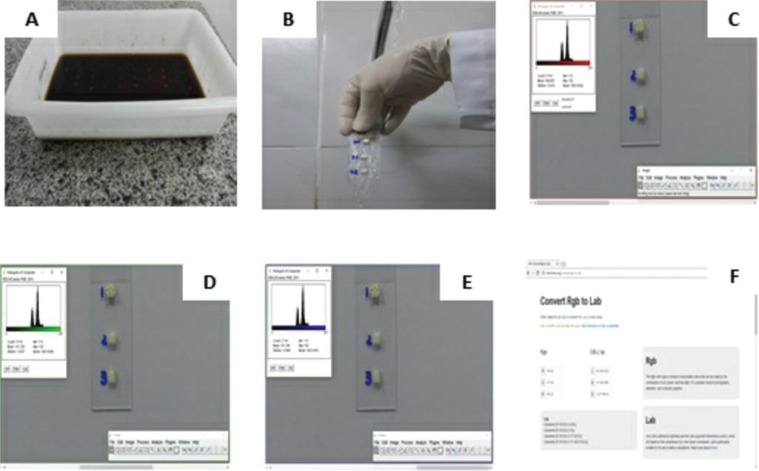
(A) The specimens were stained by full immersion of the finished slides in a coffee solution, which was renewed every 24 hours for 3 days. (B) After the period of staining, the specimens were washed in running water per 30 seconds, removing excess coffee from the surfaces. The colorimetrical analysis was made by a scientific pictures processing software (ImageJ, 32-bit, Microsoft Java) which evaluates coloration through pixels count. (C) The specimen was selected a 20 to 21 pixels area for gray/ brightness level analysis of the image pixels represented by the red-R.. (D) The specimen was selected a 20 to 21 pixels area for gray/ brightness level analysis of the image pixels represented by the green-G. (E) The specimen was selected a 20 to 21 pixels area for gray/ brightness level analysis of the image pixels represented by the blue-B. colors bands intensity, on the RGB system. (F) The data obtained were saved on a worksheet and subsequently transformed to CIELAB colors space (hthttp://colormine.org/convert/rgb-to-lab) in which the points provide more uniform numeric differences regarding visual differences.

The specimens were stained by full immersion of the finished slides in a coffee solution, which was renewed every 24 hours for 3 days (Fig. 2A). At each refill of coffee solution, prepared by mixing 500 ml of boiled water with 45g of soluble coffee (Nescafe®), colorful images were taken of specimens. After the period of staining, the specimens were washed in running water per 30 seconds (Fig. 2B), removing excess coffee from the surfaces. Subsequently, the samples were left at room temperature for a few minutes for complete drying. After drying, the final color images of the specimens were obtained.

-Data collection

The specimen’s digital pictures were carefully taken with a digital camera (Samsung CMOS, 3.264x1.836 pixels resolution, 3.70 mm focal length and 2.6 aperture. Samsung, South Korea). A white background was used and the ambient light was controlled to avoid shading, being the pictures taken at the same time of the day.

The first colorimetrical analysis was made by a scientific pictures processing software (ImageJ, 32-bit, Microsoft Java) which evaluates coloration through pixels count. On each specimen was selected a 20 to 21 pixels area for gray/ brightness level analysis of the image pixels represented by the red-R (Fig. 2C), green-G (Fig. 2D) and blue-B (Fig. 2E) colors bands intensity, on the RGB system.

The data obtained were saved on a worksheet and subsequently transformed to CIELAB colors space (hthttp://colormine.org/convert/rgb-to-lab) in which the points provide more uniform numeric differences regarding visual differences (10) (Fig. 2F).

-Colorimetric measurement

As a first method of colorimetric measurement, ImageJ software, which is free and consolidated for image processing and analysis, was developed by Wayne Rasband at the National Institute of Mental Health, USA (19).

For the samples shade colors analysis, each picture was separately open and analyzed on 3 images of distinct gray shades relating to the colors: red, green and blue respectively, and then the color average was assessed for each shade, being the result variable from 0 to 255 on the RGB scale.

In order to standardize color measurement, it was used Colormine© online software to the conversion of the RGB scale to a CIE L* ab color scale, which is the present most used for color quantitative description of an object due to its uniformity, using the following equation: CIE L* = 100* L ⁄ 255. This system is based on three elements: luminosity, tone and saturation or chromaticity. Color parameters indicate luminosity (L*) which varies from 0 (absolute black) to 100 (absolute white), the coordinate a* positions on the green axis (if negative a-) – red (if positive a+), the coordinate b* positions on the yellow axis (in negative b-) – blue (if positive b+).

-Different gel treatments

After the tooth staining period, the specimen slides were randomly divided into three groups (N = 6): WHITENESS Group (16%) (Whiteness Perfect, Joinvile, SC, Brazil), H2O2 Group (5%) (Farmafórmula, Parnaíba, PI, Brazil) and CARBOPOL GEL Group (Farmafórmula, Parnaíba, PI, Brazil) to receive bleaching procedures.

Carbamide peroxide, whitening agent present in the WHITENESS gel, is the agent most used in home bleaching, also being used in the office varying only the concentration of the active. Carbamide peroxide has many advantages, such as not requiring heat and being effective and safe with minimal side effects. H2O2 is the most used agent in office, being activated with light or heat; its results are accelerated, being more caustic, what requires a greater care in the application, since it can produce side effects greater than the effects produced by carbamide peroxide. Carbopol is an acrylic polymer with thickening function, increasing the adhesion in the dental tissues, releasing oxygen more slowly (20).

Each bleaching gel was handled and managed according to the manufacturer’s instructions. A 2mm thick layer was applied throughout the experimental area of the enamel surface of each specimen in each group.

Every 24 hours, the specimens were washed with distilled water and toothbrush for a complete removal of the gel. Subsequently, they were left at room temperature for 30 minutes for drying. Images of the specimens were obtained at each reapplication interval of the gels. The bleaching period of the groups occurred for 5 days in a hermetically sealed container with relative humidity control in 85%.

-Statistical analysis 

The data are expressed as mean ± SEM and/or median. The normality of the data was tested through the Shapiro-Wilk Test. Difference between the two groups was analyzed using Mann-Whitney U test, for no-parametric data, and test T unpaired, for parametric data.

## Results

After 48h the mean bleaching rate of the samples submitted to the WHITENESS gel was 13.6 (± 1.2), whereas the samples submitted to H2O2 presented a mean of 9.8 (± 1.4) and the samples in which only CARBOPOL GEL had a mean bleaching of 2.9 (± 0.6). After 72 hours (Fig. 1B), WHITENESS had a mean of 15.8 (± 0.7), the gel with H2O2 presented an average of 14.4 (± 1.5), and CARBOPOL GEL 4.6 (± 1.0). After completing 96h whitening, WHITENESS presented a lightening average of 18.3 (± 0.8), H2O2 cleared an average of 16.7 (± 1.4) and CARBOPOL GEL 7.3 (± 0.8). It can be seen that there was no statistically significant difference between WHITENESS and H2O2 groups, while CARBOPOL GEL group obtained a significant lower whitening action than both previous groups ( Table 1). Thus, the gel that most provided whitening was WHITENESS.

**Table 1:**
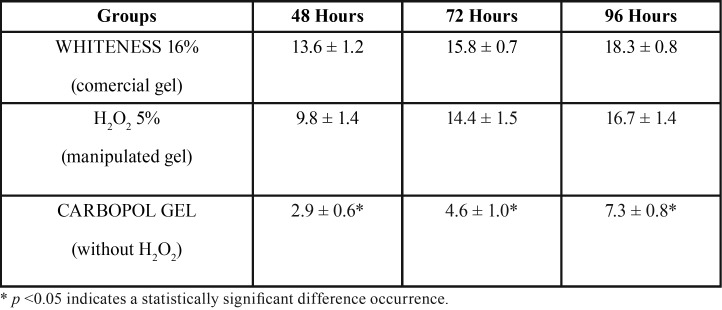
Average bleaching rate over 48 hours, 72 hours and 96 hours.

**Figure 3:**
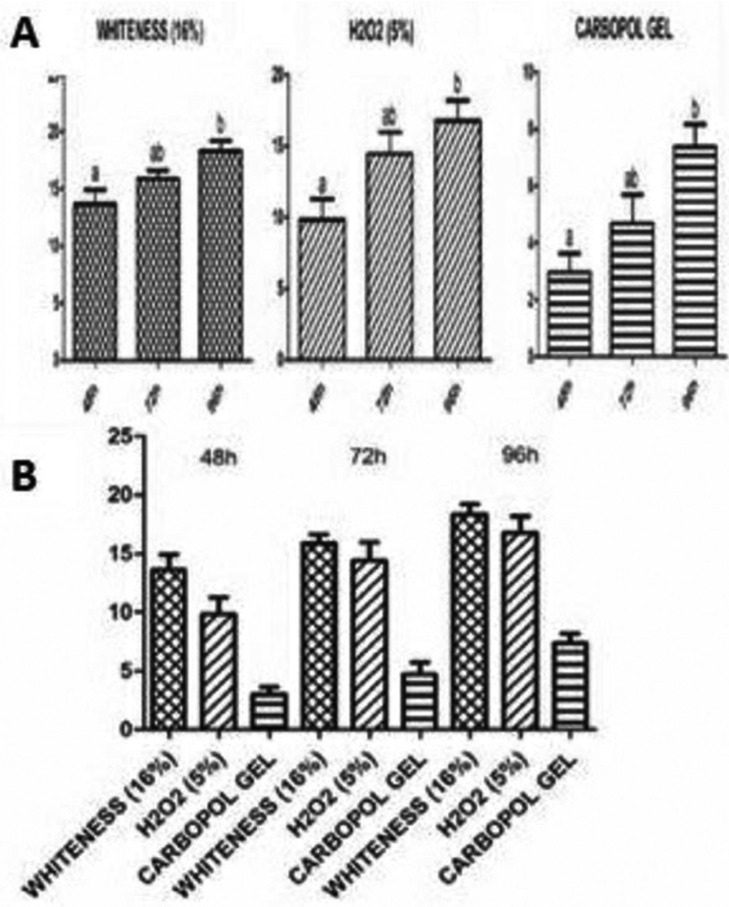
(A) Results obtained by each group in relation to time: WHITENESS, H2O2, CARBOPOL GEL. (B) Results obtained by all groups in 48h, 72h and 96h. * Same letters represent that there was no statistically significant difference between the groups, while different letters represent that there was statistically significant difference.

In all the whitening (Fig. 3A), when comparing the results obtained in 48h and 72h, there are no significant difference between the groups (represented by letter “a”), the same happens when compared the results of 72h and 96h (represented by letter “b”). However, the comparison between whitening produced in 48h and 96h, *p* <0.05 (represented by different letters), characterizing a statistically significant difference occurrence.

The whitening provided by the bleaching gels was gradual (Fig. 3B). The difference in whitening provided by WHITENESS and H2O2 was not statistically significant in all evaluated periods. When compared with H2O2 and WHITENESS both with CARBOPOL GEL, there were differences with *p* <0.05.

## Discussion

The *in vitro* dental bleaching protocol developed in this study offers a quick, inexpensive and simple methodology that allows an evaluation of tooth enamel whitening products in 96 hours.

In addition to oral health, dental aesthetics has become a priority in dental treatments. Color changes are the most common aesthetic imbalance of smile, because white teeth are considered a sign of care and beauty, which show surprising effects on self-esteem. The demand for teeth whitening has grown almost exponentially in recent years in the United States, of the 1,181 eligible surveys for analysis performed with a weighted sample of members of the American Association of Orthodontists, 88.8% of orthodontists across the country had patients who requested Bleaching (21). With the increase of aesthetic valorization and the search for whitening procedures, it is necessary to study new methodologies, which guarantee speed, practicality and accessibility, without compromising the healthy dental structure.

Previous studies (22) showed that there are similarities between human and bovine teeth, being these objects of choice for this protocol, since they are easy to obtain, without the need for approval by the Ethics Committee, which provides agility in the accomplishment of the experiment. In addition, a bovine mandible presents eight incisors of larger dimensions than the human ones, favoring the research with tests in a single element, as well as its handling and preparation. When compared to other methodologies (23-25), this protocol guarantees, in simple steps of polishing and staining, satisfactory results for evaluating dental bleaching procedures.

The color of the tooth is determined by a combination of different optical properties of enamel, dentin and pulp, staining varies according to etiology, appearance and adhesion to dental structure (26). The coloration of the dental surface by colored solutions, coffee and tea (27), beverages such as red wine (28) and blood (10), has been reported in many studies. These substances can lead to yellow and brown spots on the teeth (29). In this study, we opted for the use of coffee, because it is inexpensive, easy to prepare, and routinely used by most people. Color determination can be performed with spectrophotometers, however the technique is time-consuming and costly, since it requires a specific device (16). However, the analysis of tooth staining chosen for this protocol through digital photographs is relatively simple and sufficiently accurate to allow evaluation of the therapeutic outcome of teeth whitening procedures, although some factors affect color and brightness even performing a highly standardized methodology (30).

Using the adapted methodology (9), the photographs obtained were analyzed in ImageJ software due to its easy of manipulation and accessibility, unlike Adobe Photoshop, which is a private domain software that has tools that require user dexterity. ImageJ is an image editor compiled in Java, which allows it to run on any operating system with platform support, besides being free, its tools are practical, which gives it a large community of users in scientific research.

When analyzing the obtained results, it is observed that the groups evaluated had a gradual increase in bleaching and that the proposed protocol guarantees statistically significant results in 96 hours. Therefore, the effectiveness of the procedure performed is emphasized, considering the practicality of its handling and access, which gives it a reputation in the scientific field.

## Conclusions

The proposed protocol guarantees statistically significant results in 96 hours, confirming the efficacy, cheapness, viability and practicality of the protocol developed in this study.
